# Effect of green cardamom on the expression of genes implicated in obesity and diabetes among obese women with polycystic ovary syndrome: a double blind randomized controlled trial

**DOI:** 10.1186/s12263-022-00719-6

**Published:** 2022-12-15

**Authors:** Sahar Cheshmeh, Negin Elahi, Maysa Ghayyem, Elaheh Mosaieby, Shima Moradi, Yahya Pasdar, Susan Tahmasebi, Mehdi Moradinazar

**Affiliations:** 1 Molecular and Experimental Nutritional Medicine Department, University of Potsdam, Nuthetal, Germany; 2 Student Research Committee, Kermanshah University of Medical Sciences, Kermanshah, Iran; 3 Department of Internal Medicine, Factually of Medicine, Tehran Medical Sciences, Islamic Azad University of Medical Sciences, Tehran, Iran; 4 Department of Pathology, Charles University, Faculty of Medicine in Pilsen, Pilsen, Czech Republic; 5 Department of Nutritional Sciences, Research Center for Environmental Determinants of Health (RCEDH), Health Institute, Kermanshah University of Medical Sciences, Kermanshah, Iran; 6 Department of Biochemistry, Kermanshah University of Medical Sciences, Kermanshah, Iran; 7 Clinical Research Development Center, Imam Khomeini and Mohammad Kermanshahi and Farabi Hospitals, Kermanshah University of Medical Sciences, Kermanshah, Iran; 8 Department of Nutrition Sciences, School of Nutritional Sciences and Food Technology, Kermanshah University of Medical Sciences, Kermanshah, Iran

**Keywords:** Polycystic ovary syndrome, Green cardamom, Obesity genes, Diabetes genes

## Abstract

**Background:**

Polycystic ovary syndrome (PCOS) is an endocrine disease in which related to obesity, metabolic disorders and is considered as one of the main causes of infertility in women. This trial was investigated the effects of green cardamom on the expression of genes implicated in obesity and diabetes among obese women with PCOS.

**Methods:**

One hundred ninety-four PCOS women were randomly divided two groups: intervention (*n* = 99; 3 g/day green cardamom) and control groups (*n* = 95). All of them were given low calorie diet. Anthropometric, glycemic and androgen hormones were assessed before and after 16-week intervention. The reverse transcription-polymerase chain reaction (RT-PCR) method was used to measure fat mass and obesity-associated (FTO), peroxisome proliferative activating receptor- (PPAR-), carnitine palmitoyltransferase 1A (CPT1A), acetyl-CoA carboxylase beta (ACAB), leptin receptor (LEPR), ghrelin, and lamin A/C (LAMIN) genes expression in each group.

**Results:**

Anthropometric indices were significantly decreased after intervention in both two studied groups. Glycemic indices and androgen hormones were significantly improved in the intervention group compared to the control group. The expression levels of *FTO*, *CPT1A*, *LEPR*, and *LAMIN* were significantly downregulated compared to control group (*P* < 0.001), as well as, PPAR-y was significantly upregulated in the intervention group after intervention with green cardamom compared to control group (*P* < 0.001).

**Conclusion:**

This current study showed that the administration of green cardamom is a beneficial approach for improving anthropometric, glycemic, and androgen hormones, as well as obesity and diabetes genes expression in PCOS women under the low-calorie diet.

**Trial registration:**

This trial was registered with the Iranian Clinical Trials Registry (registration number: IRCT20200608047697N1). 1 August, 2020; https://www.irct.ir/trial/48748

## Background

Polycystic ovary syndrome (PCOS) is a common and complex endocrine disease in women and is considered one of the main causes of infertility in the reproductive years [1]. Women with PCOS often refer to medical care for menstrual disorders, clinical manifestations of hyperandrogenism, and infertility. PCOS is associated with hyperandrogenism, hyperinsulinemia, changes in the hypothalamic–pituitary–ovarian axis, ovulation disorders, and irregular menstruation, as well as mood swings, anxiety, and depression [2]. These patients are often prone to metabolic disorders characterized by weight gain and obesity, insulin resistance, type 2 diabetes, and cardiovascular disease [3]. The etiology of PCOS is not fully understood. However, some studies have found insulin resistance to be effective in its pathogenesis [4, 5]. Additionally, oxidative stress and an increase in inflammatory cytokines have also been reported to contribute to PCOS development [2].

Recent studies have found that genetic factors play a key role in the development of obesity and insulin resistance in patients with PCOS [6]. For instance, the fat mass and obesity associated (*FTO*) gene increases adipose tissue, especially in the abdomen area, as well as hyperandrogenism tends to an increase in PCOS' incidence [7–9]. Peroxisome proliferative activating receptors (*PPARs*) are part of the nuclear hormone receptors [10]. The *PPAR*-*γ* gene is vital in maintaining normal ovarian function because the *PPAR*-*γ* gene isoforms regulate metabolism, hormones related to reproduction, and ovarian function [10, 11].

Green cardamom consists of the whole dried fruit of *Elettaria cardamomum (Linn)*, which belongs to the ginger family [12]. As a seasoning, green cardamom contains polyphenols such as flavonoids (lutolin), flavonols (quercetin and camperfor), and anthocyanins, all of which have antioxidant and anti-inflammatory properties [13]. Green cardamom might affect insulin sensitivity, inflammation and liver stasis by suppressing oxidative stress [14]. So far, several studies have been conducted on the benefits of green cardamom, such as antimicrobial, anti-cancer, anti-inflammatory, and antioxidant activities in animal models [14, 15]. Interventional studies with green cardamom in humans show a reduction in metabolic and inflammatory diseases, for instance, obesity, diabetes and pre-diabetes, cardiovascular disease, and hypertension [16, 17].

Considering that most studies in this field have been conducted mostly in cell and animal models, and there are few human studies in other fields other than polycystic ovary syndrome, this current randomized clinical trial was aimed to evaluate the effects of green cardamom supplementation on the expression of genes implicated in obesity and diabetes among obese women with PCOS.

## Material and methods

### Study design

This randomized, double-blind, placebo-controlled clinical trial study was conducted to evaluate the effects of green cardamom supplementation on obesity and diabetes gene expression among obese women with PCOS referring to gynecology and female infertility clinics. CONSORT statement for randomized clinical trials [18] was used to design of the current study. According to the increase in insulin sensitivity in the previous study [19] with 95% power and 5% significance, the sample size was considered to be 70 subjects for each group. For more reassurance and possible dropouts, we entered 100 subjects in each group. The trial was ethically approved by the Ethics Committee of Kermanshah University of Medical Sciences (Ethical NO: IR.KUMS.REC.1399.375) and registered with the Iranian Clinical Trials Registry (registration number: IRCT20200608047697N1). A written consent form was completed for all subjects after explaining the objectives of the study, grant no: 990412.

### Participants, recruitment, and randomization

**Fig. 1:**
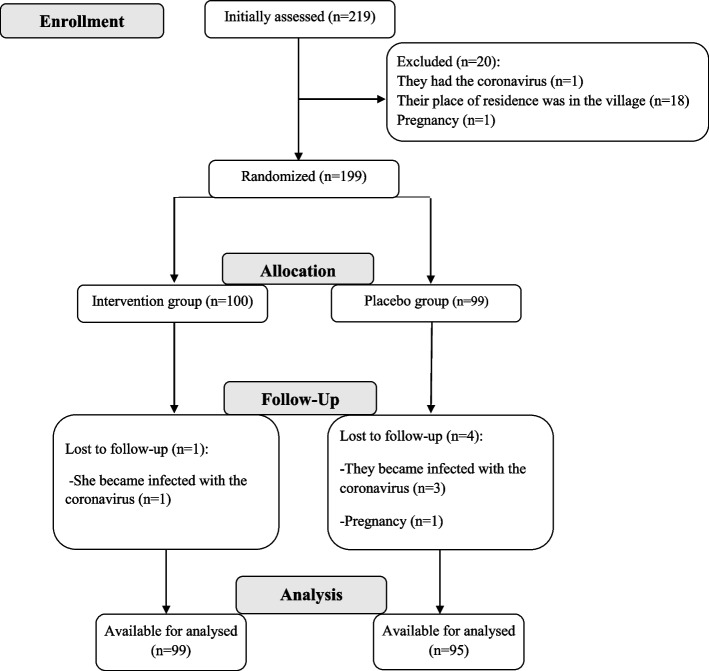
CONSORT flow chart of study

Study subjects were recruited from gynecology and female infertility clinics in Kermanshah, western Iran. Inclusion criteria Women with PCOS are diagnosed according to Rotterdam criteria if there are at least two factors: (1) oligomenorrhea or amenorrhea; (2) biochemical or clinical signs of increased androgens in the blood; and (3) having polycystic ovaries based on ultrasonography report, as well as age 18–45 years, body mass index (BMI) ≥ 30 kg/m^2^, and willingness to cooperate in this study. We did not include pregnant and lactating women, women with diseases such as autoimmune diseases, gastrointestinal, liver, thyroid, and unstable cardiovascular diseases, severe depression (due to inability to answer questions), mental illness, severe respiratory disease (asthma and chronic bronchitis), consumption of any vitamins, minerals, other dietary supplements, allergies to green cardamom, green cardamom tea, and green cardamom products. Furthermore, we did not include women receiving medications for the mentioned diseases that might interfere with green cardamom. Initially, 219 subjects were assessed. Subsequently, twenty subjects were excluded on account of the coronavirus exposure, inaccessible remote residence, and pregnant. Finally, subjects were randomly divided into two groups of placebo (*n* = 99) and intervention group (*n* = 100) using the random number table method (Fig. 1).

### Intervention

All studied subjects underwent a weight loss diet that reduced their daily calorie intake by 400–500 kcal per day based on their adjusted ideal weight. According to previous studies, the dose of cardamom powder was three grams per day. This dose of cardamom improved the lipid profile, increased total antioxidant status, decreased systolic and diastolic blood pressure, improved inflammatory markers and liver enzymes, and no toxicity was observed with this dose [17, 20]. Therefore, patients in the intervention group were given three 1000 mg cardamom capsules of Karen Company three times a day to reduce possible gastrointestinal side effects with meals. On the other hand, patients in the placebo group received three placebo tablets containing starch powder three times a day with the same shape, color, and size of cardamom supplement. Each supplement pack was coded by a representative’s company, so the researcher and the subjects were not aware of the content of the packs.

### Measurements

All subjects were asked to provide demographic information, including age and marital status. Furthermore, we collected their anthropometric indices, dietary intake, physical activity, biochemical indices, and expression of obesity and diabetes genes before and after intervention.

### Anthropometric indices

In the current study, height was measured with a wall-mounted stadiometer (Seca, Hamburg, Germany) while their shoulders, hips, and heels were in contact with the wall. The subjects’ weight and body fat mass were measured using bioelectrical impedance analysis on a body analyzer device (Inbody Co, Seoul, Korea) in standing position with light clothing and no shoes. The non-stretched and flexible tape was used to measure waist circumference (WC) at the level of the iliac crest with a precision of 0.1 cm [21]. BMI was calculated by dividing weight in kilograms by height in meters squared.

### Dietary assessment

A 3-day food record (2 days of weekdays and the weekend) was completed to evaluate the dietary intake, micro- and macronutrients, and the amount of vitamin D intake through diet before and after 16-week intervention by a trained dietitian. The energy and nutrients of their dietary intake were calculated by NUTRITIONIST IV software using the United States Department of Agriculture Food Composition Table, which was modified for Iranian foods [22].

### Physical activity

The International Physical Activity Questionnaire (IPAQ)-short form before and after the intervention was completed by an interviewer for all studied subjects. The validity and reliability of the questionnaire had previously been confirmed in Iran [23].

### Biochemical indices

At the beginning of the follicular phase (third day of the menstrual cycle), 10 cc of fasting venous blood was collected after 12 h of fasting overnight from all subjects. The blood samples were centrifuged, and the serum was stored at – 80 °C until analysis. Fasting blood sugar (FBS) was measured by an enzymatic method (Pars Azmoon Co., Iran). Fasting insulin concentrations and vitamin D3 were measured by Electrochemiluminescence (ECL) method. Glycated hemoglobin (Hb) A_1C_ was analyzed by ion exchange chromatography. The Homeostatic Model Assessment for Insulin Resistance (HOMA-IR) was calculated using the following formula:

HOMA-IR: [Fasting serum insulin (μU/ml) × fasting glucose (mmol/l)]/22.5 [24].

Follicle-stimulating hormone (FSH) and luteinizing hormone (LH) levels were measured by radioimmunoassay (RIA) technique by a LKB gamma counter. Testosterone, prolactin, thyroid stimulating hormone (TSH), androstenedione, and dehydroepiandrosterone (DHEA) were measured in these subjects by Monobinde kit and SGHB by ILB kit using ELISA device. Measurement of ghrelin by ELISA method using Human Acylated Ghrelin kit (SPI Bio Company, France). Leptin was measured based on ELISA with a dual antibody method (Sandwich) prepared by the company DRG.

### Expression of obesity and diabetes genes

Blood samples were stored in coated vials of ethylenediaminetetraacetic acid (EDTA) to evaluate the expression of *FTO*, *PPAR-γ*, carnitine palmitoyltransferase 1A (CPT1A), acetyl-CoA carboxylase beta (*ACACB*), leptin receptor (*LEPR*), ghrelin (*GHRL*), and lamin A/C (*LAMIN*) genes. Using Ficoll-Histopaque solution gradient (Ficoll-paque, Miltenyi Biotec GmbH, Germany), peripheral blood mononuclear cells (PBMC) were separated during density gradient centrifugation (Ficoll-paque, Miltenyi Biotec GmbH, Germany). Using Trisor Regaent kit (YTzol pure RNA, Iran), total RNA from PBMC cells was extracted. One microgram of the extracted RNA was applied for complementary DNA synthesis (cDNA) by Prime Script-RT reagent kits (Takara Bio Ink. Tokyo, Japan). Dedicated primers purchased from Metabion (Metabion, Germany) are presented in Table 1. The fold change formula was used to calculate the data after normalizing it to the rate of 18SrRNA expression as a housekeeping control gene. All samples were done in three versions.

**Table 1:** Primers sequences for RT-PCR amplification

**Gene name and symbol**	**Sequence (5**΄** → 3΄)**
*FTO*	F:5′-ACTTGGCTCCCTTATCTGACC-3′
	R:5′-TGTGCAGTGTGAGAAAGGCTT-3′
*CPT1A*	F: 5′-TCCAGTTGGCTTATCGTGGTG-3′
	R: 5′-TCCAGAGTCCGATTGATTTTTGC-3′
*ACACB*	F: 5′-CAAGCCGATCACCAAGAGTAAA-3′
	R: 5′-CCCTGAGTTATCAGAGGCTGG-3′
*PPAR-γ*	F:5′-GATGCCAGCGACTTTGACTC-3′
	R:5′-ACCCACGTCATCTTCAGGGA-3′
*LEPR*	F: 5′-GAATGTCATGTGCCTGTGCC-3′
	R: 5′-GGGCTGGACCACGAAATCTT-3′
*GHRL*	F: 5′-GTTGGGATCAAGTTGTCAGG-3′
	R: 5′-TGGGAGAACAGAGGTGGC-3′
*LMNA*	F: 5′-ACCAAAAAGCGCAAACTGGAG-3′
	R: 5′-GGTAAGTCAGCAAGGGATCATCT-3′
*18 s rRNA*	F:5′-ACCCGTTGAACCCCATTCGTG A-3′
	R:5′-GCCTCACTAAACCATCCAATCGG-3′

*F* forward, *R* reverse

### Statistical analysis

SPSS (SPSS Inc. Chicago, IL, USA version 19) [25] was used to analyze data from this current trial. Kolmogorov–Smirnov test was applied to the data normality. Basic characteristics of studied subjects described by mean ± standard deviation (SD), percent frequency, and chart. To compare qualitative variables, the chi-square test was used. Mann–Whitney *U*, and independent sample *t* test were used to evaluate the quantitative variables difference between the two groups. The difference in quantitative variables within the studied groups was analyzed by paired sample *t* test or Wilcoxon. Per protocol analyses were performed on only those adhering to the protocol. A significance level of less than 0.05 was considered.

## Results

One hundred and ninety-nine of the subjects fulfilled the inclusion criteria and participated in the study, but five subjects dropped out for the following reasons: one due to coronavirus infection in the intervention group, three due to coronavirus infection, and one due to pregnancy in the placebo group. Therefore, 194 subjects (intervention group 99 subjects, placebo group 95 subjects) completed the trial (Fig. 1). Ultimately, statistical analyses were performed on all 194 participants.

The mean age in the intervention and placebo groups was 32.99 ± 5.57 and 33.81 ± 5.42 years, respectively, and there was no difference between the two studied groups (*P* = 0.073). Moreover, there was no difference between the two studied groups in terms of physical activity, marital status, weight, BMI, WC, and BFM. Table 2 presents the basic characteristics of the two studied groups.

**Table 2:** Basic characteristics of studied subjects with PCOS

Variables	Intervention (*n* = 99)	Placebo (*n* = 95)	P*
Age, year	32.99 ± 5.57*	33.81 ± 5.42	0.073
Total MET, MET min/week	351.39 ± 502.01	294.76 ± 456.84	0.271
Marital status, married %	85.9	82.1	0.303
**Anthropometric indices**			
Weight, kg	86.26 ± 10	87.38 ± 10.75	0.506
WC, cm	113.01 ± 12.55	113.57 ± 7.89	0.683
BMI, kg/m^2^	34.78 ± 3.39	35.18 ± 5.16	0.989
Body fat, %	46.12 ± 2.72	47.13 ± 3.11	0.151
**Biochemical indices**			
FBS, mg/dl	106.2 ± 11.88	104.88 ± 10.87	0.33
HbA_1C_, %	6.78 ± 2.26	6.97 ± 2.12	0.425
Insulin, μU/ml	27.12 ± 3.92	26.02 ± 5.14	0.06
HOMA-IR	7.09 ± 1.28	6.72 ± 1.41	0.617
Leptin, ng/ml	28.62 ± 9.35	30.71 ± 8.45	0.324
TSH, μIU/mL	2.86 ± 0.24	2.89 ± 0.25	0.704
Gerlin, pmol/l	0.42 ± 0.03	0.39 ± 0.04	< 0.001
Androstenedione, ng/ml	2.09 ± 1.81	1.97 ± 0.32	0.121
DHEA, ng/dL	321.27 ± 62.38	363.39 ± 76.01	0.198
Prolactin, ng/ml	8.44 ± 4.22	7.78 ± 4.09	0.647
Testosterone, ng/ml	1.24 ± 0.21	1.35 ± 0.21	0.99
LH, IU/L	6.31 ± 1.41	5.94 ± 2.28	< 0.001
FSH, IU/L	1.82 ± 0.74	1.32 ± 0.84	0.283
SHBG, nm/l	27.77 ± 10.63	33.12 ± 10.17	0.726
25 (OH) D3, ng/mL	20.03 ± 11.23	18.7 ± 11.26	0.371

^*^Mean ± SD

P* was obtained chi-square, Mann–Whitney *U*, and independent sample *t* test

The mean of the calorie and nutrient did not differ between the two studied groups before and after the intervention. According to the given low calorie diet to all subjects, the calorie and nutrient had differences within each of the studied groups before and after intervention (Table 3).

**Table 3:** Energy and nutrients intake of subjects with polycystic ovarian syndrome

Variables	Intervention (*n* = 99)			Placebo (*n* = 95)			P2	P3
	Before	After	P1	Before	After	P1		
Energy (kcal/day)	3424.24 ± 769.51*	2216.61 ± 590.47	< 0.001	3101.48 ± 748.6	2189.48 ± 633.14	< 0.001	0.5	0.801
Protein (g/day)	121.03 ± 37.11	84.81 ± 24.01	< 0.001	106.68 ± 32.09	84.22 ± 25.21	< 0.001	0.158	0.91
Carbohydrate (g/day)	500.17 ± 140.71	348.57 ± 102.75	< 0.001	460.17 ± 138.14	342.79 ± 113.71	< 0.001	0.704	0.591
Fat (g/day)	106.82 ± 33.75	56.13 ± 18.54	< 0.001	93.91 ± 28.19	55.89 ± 20.92	< 0.001	0.193	0.639
Vitamin D (IU/day)	2.77 ± 5.37	2.32 ± 2.38	0.402	1.93 ± 3.81	2.11 ± 2.34	0.098	0.043	0.66

^*^All presented values are means ± SD

P1: *P* values denote significance of within-group changes

P2: *P* values denote significance of between-group difference in the baseline

P3: *P* values denote significance of between-group difference after intervention

^*^Significant difference within group throughout the study (*P* < 0.05, paired samples *t* test or Wilcoxon)

^*^Significant difference between groups throughout the study (*P* < 0.05, independent samples *t* test or *U* Mann–Whitney

After the intervention, all participating women underwent re-ultrasound. PCOS was improved in terms of cyst size and number, decreasing 54.1% in the intervention group and 35.5% in the placebo group, which was significantly different between the two studied groups (*P* = 0.031).

Table 4 showed that the mean of weight, BMI, WC, BFM, and FBS were significantly decreased after intervention in both of the two studied groups. Also, we observed that HbA_1c_, insulin, HOMA-IR, leptin, androstenedione, DHEA, and LH were significantly decreased in intervention group after intervention with green cardamom, as well as FSH, were significantly increased in this group.

**Table 4:** Anthropometric indices, glycemic indices, and androgen hormones of subjects with polycystic ovarian syndrome

Variables	Intervention (*n* = 99)		P1	Placebo (*n* = 95)		P1	P2
	Before	After		Before	After		
**Weight, kg**	86.26 ± 10	79.65 ± 10.98	< 0.001	87.38 ± 10.75	81.48 ± 12.40	< 0.001	0.156
**WC, cm**	113.01 ± 12.55	106.30 ± 9.33	< 0.001	113.57 ± 7.89	108.18 ± 9.88	< 0.001	0.366
**BMI, kg/m**^**2**^	34.78 ± 3.39	32.13 ± 4.46	< 0.001	35.18 ± 5.16	32.86 ± 5.95	< 0.001	0.015
**Body fat, %**	46.12 ± 2.72	44.48 ± 2.53	< 0.001	47.13 ± 3.11	44.76 ± 3.06	< 0.001	0.036
**FBS, mg/dl**	106.2 ± 11.88	99.28 ± 15.78	< 0.001	104.88 ± 10.87	100.68 ± 9.97	< 0.001	0.012
**HbA**_**1C**_**, %**	6.78 ± 2.26	5.59 ± 1.99	< 0.001	6.97 ± 2.12	6.67 ± 1.92	0.202	0.821
**Insulin, pmol/L**	27.12 ± 3.92	23.49 ± 4.35	< 0.001	26.02 ± 5.14	26.56 ± 5.93	0.647	0.001
**HOMA-IR**	7.09 ± 1.28	5.72 ± 1.32	< 0.001	6.72 ± 1.41	6.63 ± 1.63	0.315	0.025
**Leptin, ng/ml**	28.62 ± 9.35	20.46 ± 6.74	< 0.001	30.71 ± 8.45	29.22 ± 7.67	0.262	0.143
**Gerlin, pmol/l**	0.42 ± 0.03	0.42 ± 0.03	0.769	0.39 ± 0.04	0.41 ± 0.03	0.013	0.07
**TSH, μIU/mL**	2.86 ± 0.21	2.88 ± 0.27	0.469	2.89 ± 0.25	2.89 ± 0.34	0.787	0.724
**Androstenedione, ng/ml**	2.09 ± 1.81	1.68 ± 0.25	< 0.001	1.97 ± 0.32	1.85 ± 0.24	0.013	< 0.001
**DHEA, ng/dL**	321.27 ± 62.38	282.97 ± 64.54	< 0.001	363.39 ± 76.01	383.06 ± 48.71	0.109	0.033
**Prolactin, ng/ml**	8.44 ± 4.22	8.54 ± 3.87	0.884	7.78 ± 4.09	7.98 ± 3.65	0.747	0.594
**Testosterone, ng/ml**	1.24 ± 0.21	1.19 ± 0.3	0.188	1.35 ± 0.21	1.35 ± 0.25	0.968	0.016
**LH, IU/L**	6.31 ± 1.41	3.36 ± 1.41	< 0.001	5.94 ± 2.28	5.95 ± 1.84	0.774	< 0.001
**FSH, IU/L**	1.82 ± 0.74	2.77 ± 1.42	< 0.001	1.32 ± 0.84	1.43 ± 0.95	0.768	< 0.001
**SHBG, nm/l**	27.77 ± 10.63	29.42 ± 10.51	0.261	33.12 ± 10.17	34.79 ± 10.59	0.251	0.829
**25 (OH) D3, ng/mL**	20.03 ± 11.23	20.52 ± 10.08	0.587	18.7 ± 11.26	22.15 ± 12.79	0.06	0.68

^*^All presented values are means ± SD

P1: *P* values denote significance of within-group changes

P2: *P* values denote significance of between-group difference after intervention

^*^Significant difference within group throughout the study (*P* < 0.05, paired samples *t* test or Wilcoxon)

^*^Significant difference between groups throughout the study (*P* < 0.05, independent samples *t* test or *U* Mann–Whitney)

**Fig. 2:**
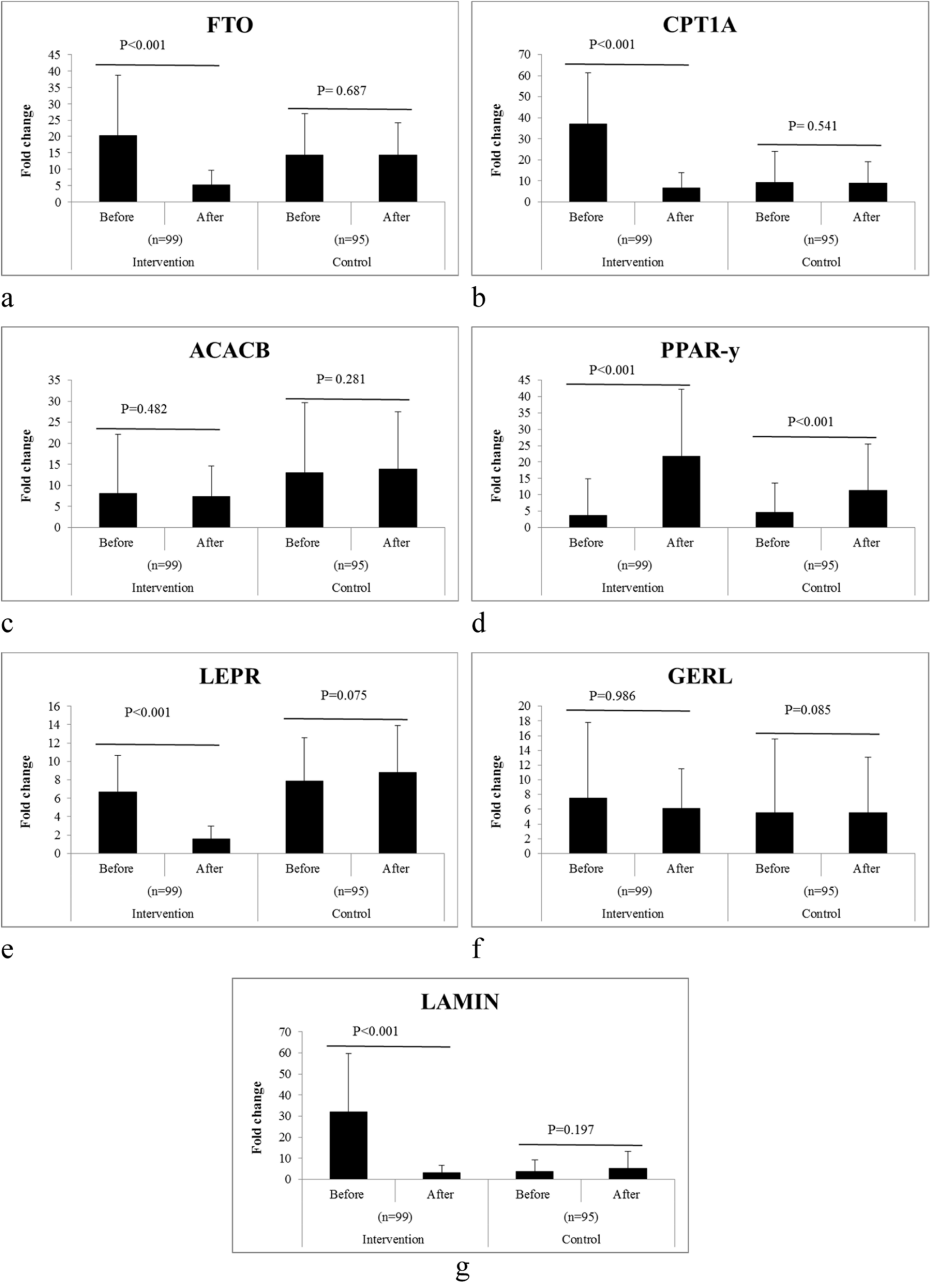
Changes of the obesity and diabetes genes including: **a** FTO, **b** CPT1A, **c** ACACB, **d** PPAR-y, **e** LEPR, **f** GERL, and **g** LAMIN before and after intervention with green cardamom

Figure 2 indicates the expression levels of the obesity and diabetes genes in both two studied groups. Among the measurement of the obesity and diabetes genes, the expression level of *FTO*, *CPT1A*, *LEPR*, and *LAMIN* were significantly downregulated in the intervention group after intervention with green cardamom (*P* < 0.001). Furthermore, *PPAR*-*γ* was significantly upregulated in this group (*P* < 0.001).

## Discussion

In this study, three gram per day green cardamom supplementation with a low-calorie diet in PCOS obese women resulted in an improvement in glycemic indices, including FBS, HbA1c, insulin, and HOMA-IR compared with placebo intake. In addition, when compared to placebo intake, androgen hormones and ultrasonography reports of ovarian function were improved in the intervention group. Furthermore, we observed that green cardamom intervention in PCOS women was associated with decreased expression of *FTO, CPT1A, LEPR*, and *LAMIN*, but *PPAR*-*γ* was considerably upregulated in this group.

PCOS is a complex disease with genetic and environmental components, and genes related to obesity and insulin metabolism appear to be involved in the etiology of this syndrome [26]. Insulin resistance and hyperinsulinemia affect 65–70% of women with PCOS, and obesity also accelerates the clinical manifestations of this syndrome in susceptible women [1].

In the current study, weight, BMI, WC, and BFM were significantly decreased in both of the two studied groups under the low calorie diet. Furthermore, all glycemic indices, including FBS, HbA_1c_, insulin, and HOMA-IR were significantly improved after 16 weeks intervention. Yaghooblou et al. [27] in their trial on pre-diabetic women, they observed that after 2 months of intervention with 3 g of cardamom, weight, BMI, WC, and insulin sensitivity were significantly decreased compared to the control group. However, other glycemic indices, including FBS, insulin, and HOMA-IR, had not changed after intervention. Another trial by Aghasi et al. [28] showed that HbA_1c_, insulin, and HOMA-IR were significantly decreased after green cardamom supplementation. For ethical consideration, we gave both groups the low calorie diet. Therefore, it seems changes in anthropometric indices after intervention in both groups are normal. Green cardamom is rich in flavonoids and isoflavones that contribute in reducing insulin resistance by decreasing adipose tissue storage [13, 14].

Our results indicated that after intervention with the green cardamom, endocrine outcomes including leptin, androstenedione, DHEA, and LH were significantly reduced in the intervention group, as well as, FSH were significantly increased in this group. A literature review of 33 studies showed a decrease in LH, prolactin, insulin, and testosterone after the administration of herbal medicine to women with PCOS [29]. In a study on overweight and obese PCOS women, Khorshidi et al. [6] reported that after quercetin supplementation, the levels of LH, testosterone, and SHBG were significantly decreased. Obesity, especially abdominal obesity, as well as insulin resistance, exacerbate hyperandrogenism. Obesity is mainly associated with increased levels of free fatty acids (FFA), which increase FFA, reducing insulin sensitivity [30]. Finally, abdominal obesity and insulin resistance synergistically affect the production of androgen hormones [1]. On the other hand, increasing adipose tissue causes the production of the hormone leptin. Leptin is a hormone encoded by the obesity gene (LPER) on human chromosome 7 [31]. High levels of this hormone are seen in some women with PCOS, which prevents the conversion of androgens to estrogen and subsequent follicular atresia [1, 31]. Therefore, it seems that green cardamom, with anti-inflammatory properties and reduced fat storage, has beneficial effects in improving the status of androgen hormones.

**Fig. 3:**
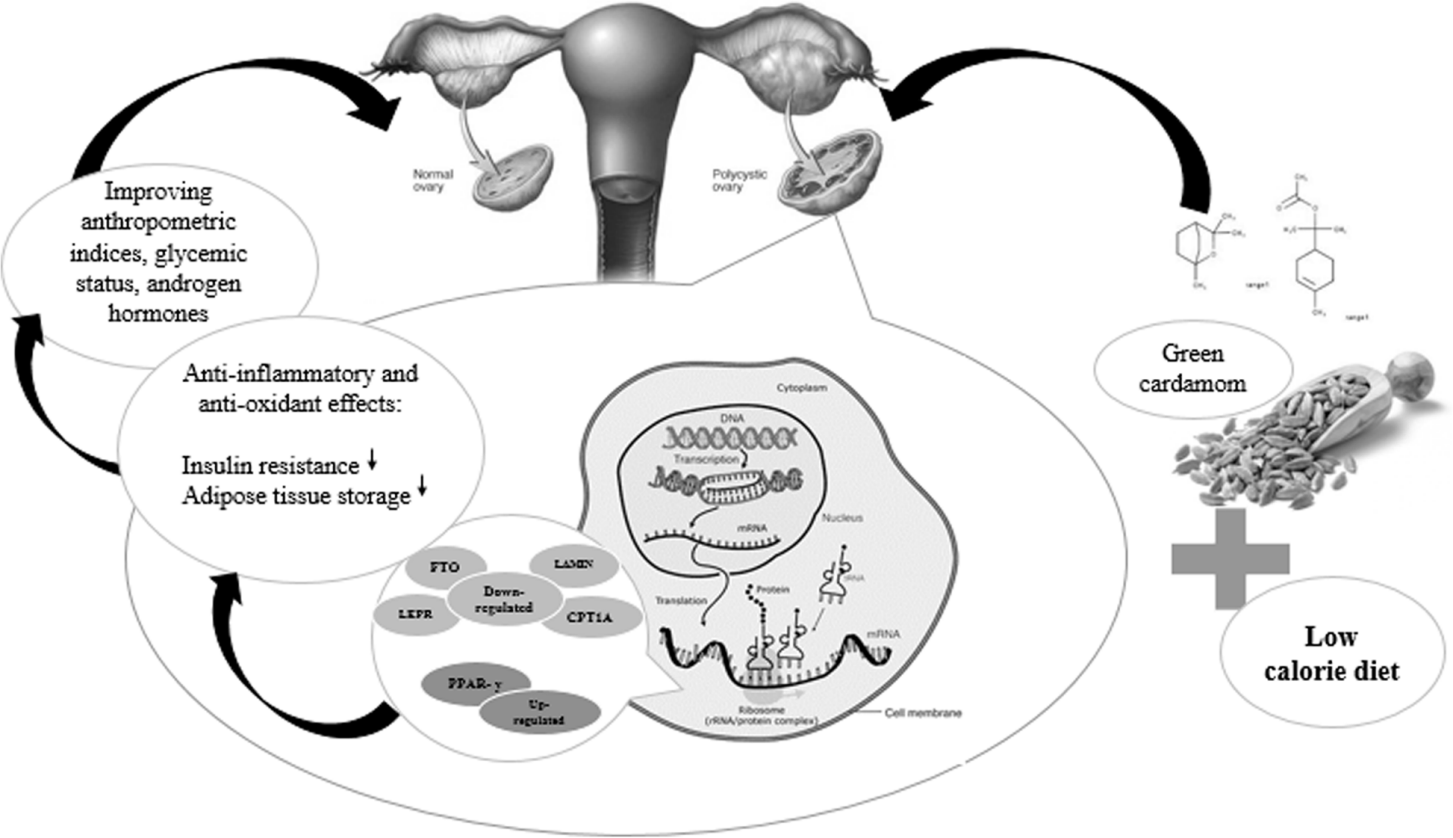
The effects of the green cardamom on obesity and diabetes genes expression

The current study found that after a green cardamom intervention, *FTO*, *CPT1A*, *LEPR*, and *LAMIN* were downregulated while PPAR-*γ* was upregulated in PCOS women. Limited data are available evaluating the effects of the green cardamom on obesity and diabetes genes expression (Fig. 3).

Results of a meta-analysis by Liu et al. showed that the expression level of *FTO* gene was related to a higher risk of PCOS in which the *FTO* gene appears to be involved in the pathogenesis of PCOS by increasing fat mass and eventually obesity [32]. In animal models, the expression level of *CPT1A* was associated with increasing BMI, WC, and hypertriglyceridemia [33, 34]. It has been established that *LEPR* polymorphism is linked to obesity, insulin resistance, and dyslipidemia, and that serum leptin in PCOS women is elevated due to a high quantity of adipose tissue [35, 36]. Excessive production of inflammatory markers in adipose tissue is mediated by the *LAMIN* gene (mapped on the long arm of chromosome 1) through macrophages, which leads to diabetes development [37]. Nasri et al. [38] discovered that administering omega three fatty acids with anti-inflammatory properties could up-regulate PPAR-γ in PCOS women (*P* = 0.005) in a randomized clinical trial. Heshmati et al. [39] in their study showed that 4.5 g/day curcumin supplementation was related to *PPAR*-*γ* coactivator 1a gene up-regulation in PCOS women (*P* = 0.011). Similarly, Daneshi et al. [17] reported that 3 g/day cardamom supplementation could increase the level of Irisin, which can improve *PPAR*-γ coactivator 1a secretion in overweight and obese with non-alcoholic fatty liver disease patients. The *PPAR*-*γ* gene has a role in regulating metabolism, reproductive hormones, and ovarian function [10, 11]. Due to its anti-inflammatory and antioxidant properties, green cardamom plays an important role in reducing inflammation and improving insulin resistance [14]. Our other study showed that green cardamom consumption was associated with decreased levels of inflammatory factors and down regulation genes [40].

In conclusion, this is the first study to evaluate the effect of green cardamom supplementation on obesity and diabetes gene expression in PCOS women.

This study demonstrated that a green cardamom intervention improved anthropometric indices, glycemic indices, and sexual hormones, as well as the expression level of obesity and diabetes genes *FTO, CPT1A, LEPR, LAMIN*, and *PPAR-γ* genes in PCOS women.

## Data Availability

Data will be available upon request from the corresponding author.

## Abbreviations

ACACB: Acetyl-CoACarboxylase Beta
BMI: Body mass index
*CPT1A*: Carnitine palmitoyltransferase 1A
DHEA: Dehydroepiandrosterone
EDTA: Ethylenediaminetetraacetic acid
FTO: Fat mass and obesity associated
FBS: Fasting blood sugar
FSH: Follicle-stimulating hormone
GHRL: Ghrelin
(Hb) A_1C_: Glycated hemoglobin
HOMA-IR: Homeostatic Model Assessment for Insulin Resistance
IPAQ: International Physical Activity Questionnaire
LAMIN: Lamin A/C
LEPR: Leptin receptor
LH: Luteinizing hormone
PBMC: Peripheral blood mononuclear cells
PPARs: Peroxisome proliferative activating receptors
PCOS: Polycystic ovary syndrome
SD: Standard deviation
TSH: Thyroid stimulating hormone
WC: Waist circumference
