# Correction: Effect of green cardamom on the expression of genes implicated in obesity and diabetes among obese women with polycystic ovary syndrome: a double blind randomized controlled trial

**DOI:** 10.1186/s12263-023-00720-7

**Published:** 2023-01-27

**Authors:** Sahar Cheshmeh, Negin Elahi, Maysa Ghayyem, Elaheh Mosaieby, Shima Moradi, Yahya Pasdar, Susan Tahmasebi, Mehdi Moradinazar

**Affiliations:** 1 Molecular and Experimental Nutritional Medicine Department, University of Potsdam, Nuthetal, Germany; 2 Student Research Committee, Kermanshah University of Medical Sciences, Kermanshah, Iran; 3 Department of Nutrition Sciences, School of Nutritional Sciences and Food Technology, Kermanshah University of Medical Sciences, Kermanshah, Iran; 4 Department of Internal Medicine, Factually of Medicine, Tehran Medical Sciences, Islamic Azad University of Medical Sciences, Tehran, Iran; 5 Department of Pathology, Charles University, Faculty of Medicine in Pilsen, Pilsen, Czech Republic; 6 Department of Nutritional Sciences, Research Center for Environmental Determinants of Health (RCEDH), Health Institute, Kermanshah University of Medical Sciences, Kermanshah, Iran; 7 Department of Biochemistry, Kermanshah University of Medical Sciences, Kermanshah, Iran; 8 Clinical Research Development Center, Imam Khomeini and Mohammad Kermanshahi and Farabi Hospitals, Kermanshah University of Medical Sciences, Kermanshah, Iran

**Correction: Genes & Nutrition 17, 17 (2022)**

**https://doi.org/10.1186/s12263-022-00719-6**

Following publication of the original article [1], it came to the authors’ attention that the affiliations information had been incorrectly ordered in the authorship list and the author details section. The published article has since been corrected and the correct affiliations information may be seen in this erratum.
